# Supplementary material for: Development of a model estimating root length density from root impacts on a soil profile in pearl millet (Pennisetum glaucum (L.) R. Br). Application to measure root system response to water stress in field conditions
Source: PLoS One. 2019 Jul 22;14(7):e0214182. doi: 10.1371/journal.pone.0214182 (PMC6645461; doi:10.1371/journal.pone.0214182)
Supplement: S1 Fig — (PDF) [file pone.0214182.s001.pdf]

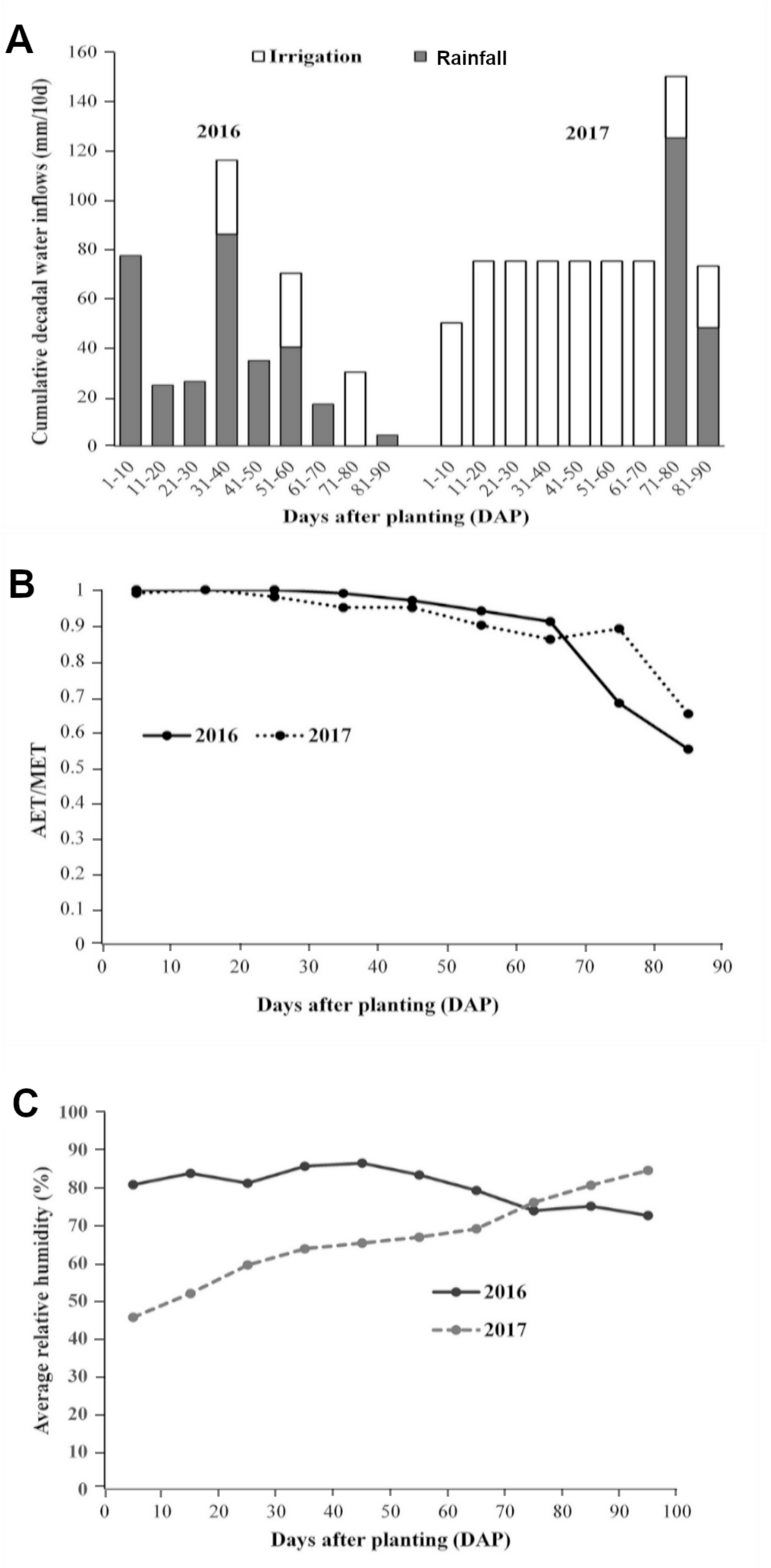

S1 Fig . Climatic data for Exp1. & 2. (A) Decadal distribution of rainfall and irrigation4 during the calibration trial in 2016 and validation trial in 2017 , (B) crop water requirement satisfaction rate (Actual evapotranspiration / Maximum evapotranspiration), and (C) average ralative air humidity (%) during the trials.
