# Supplementary material for: Development of a model estimating root length density from root impacts on a soil profile in pearl millet (Pennisetum glaucum (L.) R. Br). Application to measure root system response to water stress in field conditions
Source: PLoS One. 2019 Jul 22;14(7):e0214182. doi: 10.1371/journal.pone.0214182 (PMC6645461; doi:10.1371/journal.pone.0214182)
Supplement: S2 Fig — (PDF) [file pone.0214182.s002.pdf]

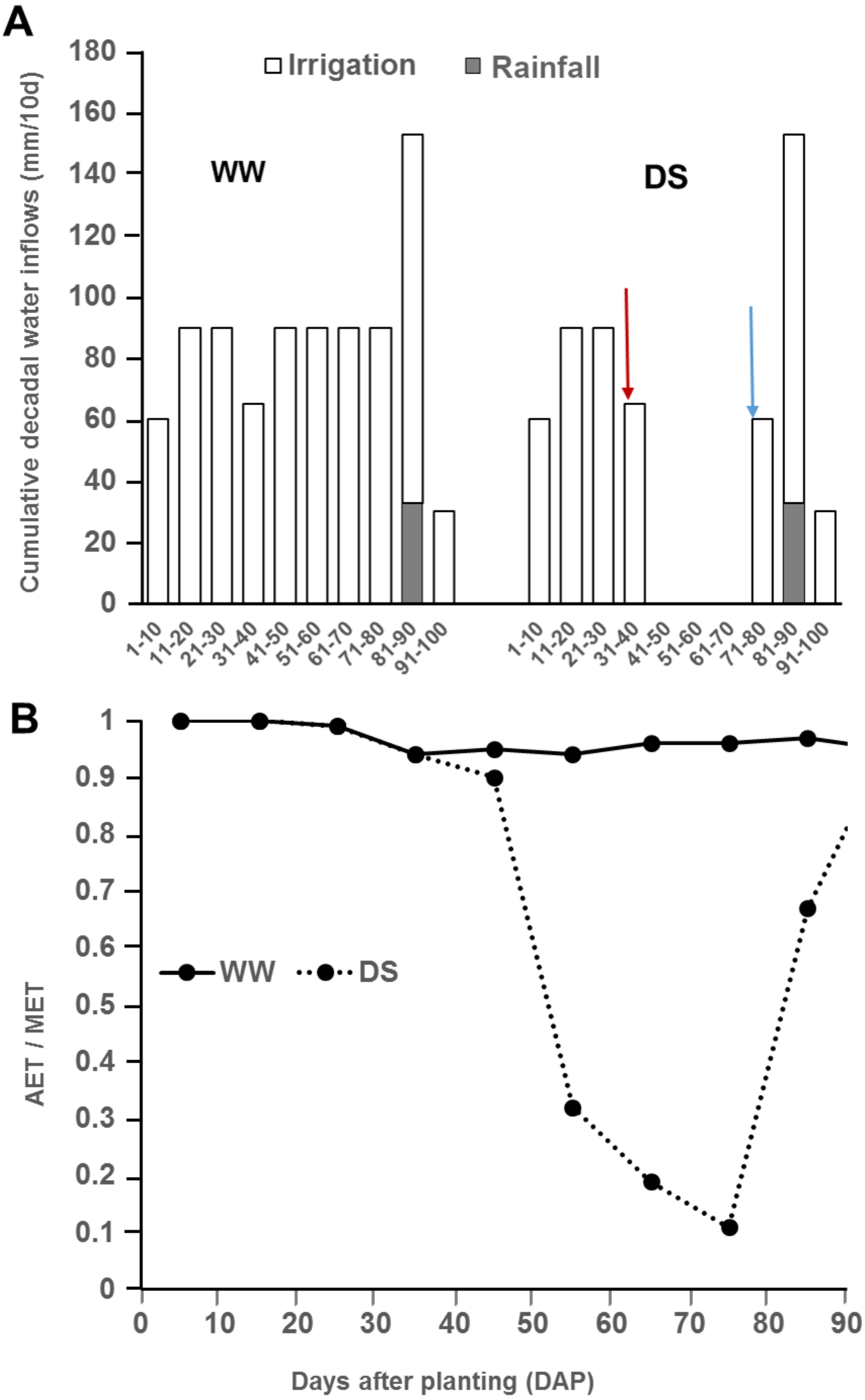

S2 Fig. Climatic data for Exp 3. (A) Decadal distriution of rainfall and irrigation for well watered (WW) and drought stress conditions (WS) , (B) Crop water requirement satisfaction rate (Actual Evapotranspiration / Maximum Evapotraspiration). The red and blue arrows represent the start and end dates of water stress application respectively.
