# Supplementary material for: Development of a model estimating root length density from root impacts on a soil profile in pearl millet (Pennisetum glaucum (L.) R. Br). Application to measure root system response to water stress in field conditions
Source: PLoS One. 2019 Jul 22;14(7):e0214182. doi: 10.1371/journal.pone.0214182 (PMC6645461; doi:10.1371/journal.pone.0214182)
Supplement: S3 Fig — (PDF) [file pone.0214182.s003.pdf]

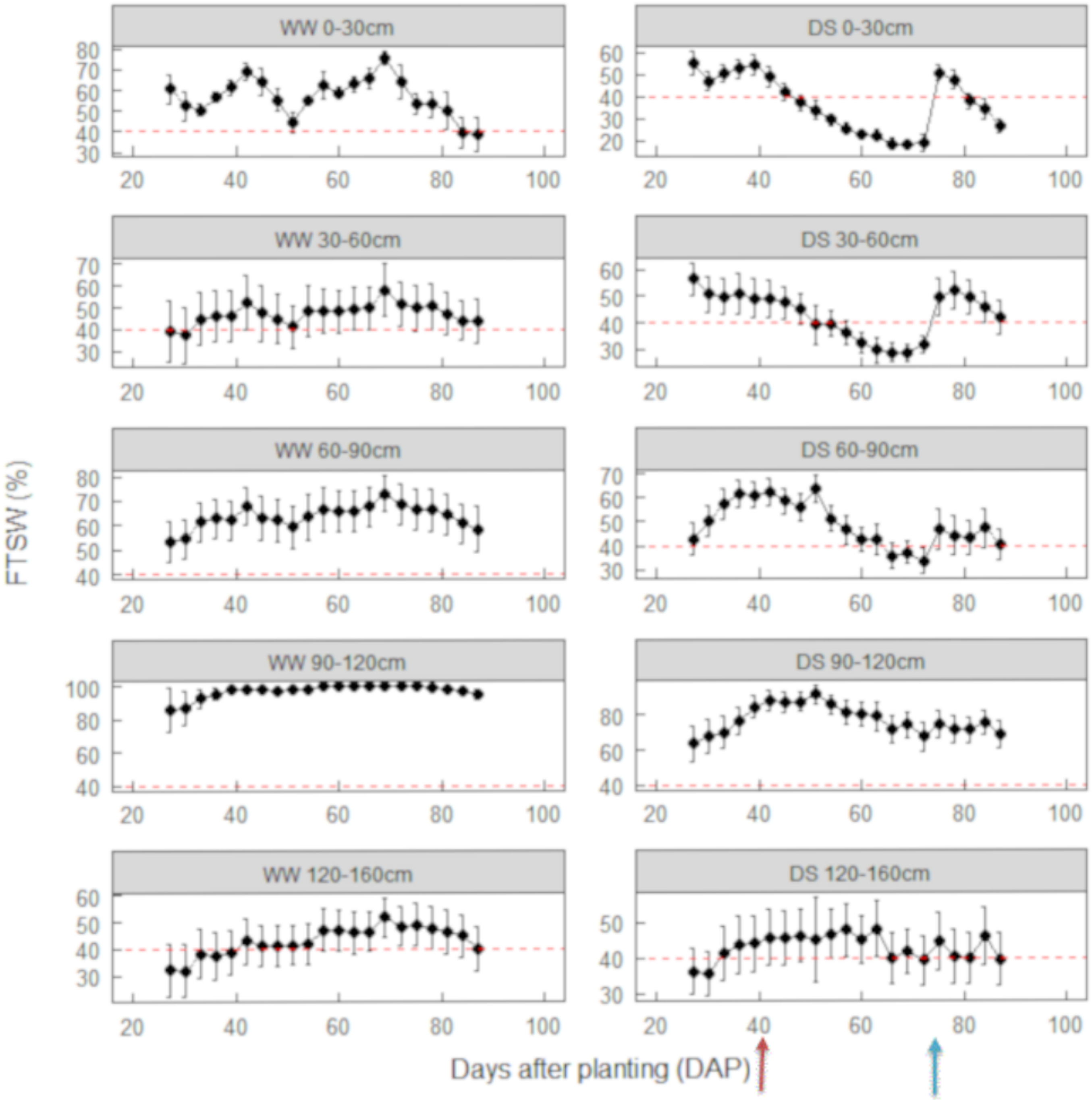

S3 Fig. Soil water content for Exp 3. Evolution of Fraction of Transpirable Soil Water variations under well-watered (WW) and dorught stress (DS) conditions. The red and blue arrows represent the start and end dates of water stress application respectively.
