# Supplementary material for: Development of a model estimating root length density from root impacts on a soil profile in pearl millet (Pennisetum glaucum (L.) R. Br). Application to measure root system response to water stress in field conditions
Source: PLoS One. 2019 Jul 22;14(7):e0214182. doi: 10.1371/journal.pone.0214182 (PMC6645461; doi:10.1371/journal.pone.0214182)
Supplement: S1 Table — (DOCX) [file pone.0214182.s004.docx]

| Clay | 2.70% |
| --- | --- |
| Silt | 3% |
| Sand | 94.50% |
| H_2_O | 7.7 |
| Potassium chloride (KCl) | 7.6 |
| NO_3_ (mg/kg) | 2.55 |
| NH_4_ (mg/kg) | 5.66 |
| N | 0.04% |
| C | 0.69% |
| C/N | 16 |
| P total (mg/kg) | 138 |
| P assimilable (mg/kg) | 18 |
| Ca^2+^  (Cmol/kg) | 3.65 |
| Mg^2+^  (Cmol/kg) | 1.44 |
| Na^+^  (Cmol/kg) | 0.10 |
| K^+^  (Cmol/kg) | 0.22 |

**S1 Table. Typical soil characteristics at the CNRA station (Bambey, Senegal).**
