# Supplementary material for: Development of a model estimating root length density from root impacts on a soil profile in pearl millet (Pennisetum glaucum (L.) R. Br). Application to measure root system response to water stress in field conditions
Source: PLoS One. 2019 Jul 22;14(7):e0214182. doi: 10.1371/journal.pone.0214182 (PMC6645461; doi:10.1371/journal.pone.0214182)
Supplement: S2 Table — (DOCX) [file pone.0214182.s005.docx]

**S2 Table. Student t-test on preferential orientation indices** (P) of fine, thick and all roots of vertical faces (v) according to the factors of the experiment

|  | **Varieties** | **Date (DAP)** | **Sampling distances to the plant** |  |  |  |  |  |  | **Depth(m)** |  |
| --- | --- | --- | --- | --- | --- | --- | --- | --- | --- | --- | --- |
| **P_f_** | 0.1111 | 0.3623 | 0.4755 |  |  |  |  |  |  | 0.2118 |  |
| **P_t_** | 0.9056 | 0.945 | **0.04518 *** |  |  |  |  |  |  | **0.001043 **** |  |
| **P_a_** | 0.1841 | 0.4088 | 0.2142 |  |  |  |  |  |  | **0.004909 **** |  |

The probability value in bold is significant at the 5% threshold
